# Phase and Structure Behavior vs. Electromechanical Performance of Electrostrictive P(VDF-HFP)/ZnO Composite Nanofibers

**DOI:** 10.3390/polym13152565

**Published:** 2021-07-31

**Authors:** Nikruesong Tohluebaji, Panu Thainiramit, Chatchai Putson, Nantakan Muensit

**Affiliations:** 1 Faculty of Science and Technology, Princess of Naradhiwas University, Narathiwat 96000, Thailand; nikruesong.t@pnu.ac.th; 2 Division of Physical Science (Physics), Faculty of Science, Prince of Songkla University, Songkhla 90112, Thailand; panu.t@psu.ac.th (P.T.); nantakan.m@psu.ac.th (N.M.); 3 Center of Excellence in Nanotechnology for Energy (CENE), Songkhla 90112, Thailand

**Keywords:** electrostrictive, electromechanical properties, poly(vinylidene fluoride-*co*-hexafluoropropylene), zinc oxide, nanofibers

## Abstract

In this work, we improved the electromechanical properties, electrostrictive behavior and energy-harvesting performance of poly(vinylidenefluoridene-hexafluoropropylene) P(VDF-HFP)/zinc oxide (ZnO) composite nanofibers. The main factor in increasing their electromechanical performance and harvesting power based on electrostrictive behavior is an improved coefficient with a modified crystallinity phase and tuning the polarizability of material. These blends were fabricated by using a simple electrospinning method with varied ZnO contents (0, 5, 10, 15 and 20 wt%). The effects of the ZnO nanoparticle size and content on the phase transformation, dielectric permittivity, strain response and vibration energy harvesting were investigated. The characteristics of these structures were evaluated utilizing SEM, EDX, XRD, FT-IR and DMA. The electrical properties of the fabrication samples were examined by LCR meter as a function of the concentration of the ZnO and frequency. The strain response from the electric field was observed by the photonic displacement apparatus and lock-in amplifier along the thickness direction at a low frequency of 1 Hz. Moreover, the energy conversion behavior was determined by an energy-harvesting setup measuring the current induced in the composite nanofibers. The results showed that the ZnO nanoparticles’ component effectively achieves a strain response and the energy-harvesting capabilities of these P(VDF-HFP)/ZnO composites nanofibers. The electrostriction coefficient tended to increase with a higher ZnO content and an increasing dielectric constant. The generated current increased with the ZnO content when the external electric field was applied at a vibration of 20 Hz. Consequently, the ZnO nanoparticles dispersed into electrostrictive P(VDF-HFP) nanofibers, which offer a large power density and excellent efficiency of energy harvesting.

## 1. Introduction

Electroactive polymers (EAPs) are intelligent materials that change their shape in response to an external electrical stimulus and exhibit great deformation [1]. In general, EAPs can be classified into two groups: the first is electronic EAPs, which are driven by an electric field; and the second is ionic EAPs, which are driven by the mobility or diffusion of ions in substances. Hence, in this study, the electrostrictive polymer regarding electronic EAPs is presented as the attractive type for the intelligent system. It can convert mechanical energy into electrical energy for energy-harvesting applications; otherwise, it can produce a field-induced strain when an external electric field is applied. The EAPs have also been applied in electromechanical utilization for functional actuators and sensors, e.g., acoustic flexible machines [2], vibration control sensors [3], smart sportswear [4], smart garments [5], and robotics [6,7]. Moreover, it possesses several benefits based on its properties such as its high mechanical strain, fast response speed, high energy density, low cost, and light weight, as well as the fact it can easily generate electricity [8,9].

The poly(vinylidenefluoridene-hexafluoropropylene) P(VDF-HFP) copolymer is one of the most significant electronic EAPs due to its high polarizability in the polymer matrix. In consideration of the fact that the electrostrictive P(VDF-HFP) can produce both sizeable mechanical deformation and electrical polarizations, it can convert between electrical and mechanical energy forms. It can be used in actuators and energy-harvesting applications [10,11]. Moreover, the P(VDF-HFP) copolymer exhibits good chemical resistance, higher solubility, flexibility, durability, lightweight, and strong hydrophobicity [6]. In addition, the incorporation of HFP groups increases the fluorine content, which leads to the higher polarizability and hydrophobicity of P(VDF-HFP) [7]. It has four phases (e.g., α, β, γ, and δ). However, in this work, the β-phase was selected as the main material phase to improve for proper electrical and mechanical properties. For the β-phase structure, its dipole moment is oriented in the same direction; the phase is an immense polarization per unit cell better than others. The β-phase has superior performance in dielectric properties, electrostrictive, pyroelectric, and ferroelectric aspects. 

According to previous literature, increasing the electrostrictive coefficient (M_33_) can offer significant electromechanical responses [12,13]. The dielectric constant and Young’s modulus are significant with a coefficient corresponding to electric polymers. To enhance the ability of the electromechanical response, we need to accrue the dielectric constant and maintain a low Young’s modulus. For fabricated techniques, there are more conventional techniques for fabricating the β-phase P(VDF-HFP). This involves mixing and blending with groups of fillers, such as montmorillonite (MMT) [14], magnesium chloride hexahydrate (MgCl_2_·6H_2_O) [15], multiwalled carbon nanotubes (MWNTs) [16], carbon black [17], WO_3_·H_2_O [18], nickel chloride hexahydrate salt [19], Ce^3+^/graphene [20] and Ni-doped ZnO/P(VDF-HFP) [21]. Parangusan et al. reported that a 0.5 wt% Ni-doped ZnO/P(VDF-HFP) nanocomposite showed the β-phase and the high dielectric constant up to 55 at 1 Hz, which achieved a maximum output voltage of 1.2 V. Sajad et al. [22] found that the electrospinning process could enhance the β-phase, and thus, adding 15% ZnO nano-powder/PVDF composite nanofibers increased the electrical response to 1.1 V. Yuennan et al. [15] presented that the large dielectric constant of a P(VDF-HFP)/MgCl_2_·6H_2_O film is up to 13 and 19 from the P(VDF-HFP)/AlCl_3_·6H_2_O film at 100 Hz. In some filler based on terpolymer content, P(VDF-TrFE-CFE) [23] has an enormous power density (7200 nW/cm^3^) at an electric field of 5 V/μm, but this terpolymer has a great modulus, requiring a complicated preparation, and it is also more expensive than an electrostrictive P(VDF-HFP) matrix.

To improve the composite polymer filled with nano-sized inorganic filler, varying the loading level of the nanofiller is an easy way to tune the electrical properties of EAP materials [24,25]. Zinc oxide (ZnO) is an excellent inorganic nanoparticle. It has high crystallinity property, thermal resistance, chemical stability, a long shelf life, is eco-friendly and compatible with dispersion in varieties of polymer matrices. Moreover, ZnO shows the piezoelectric properties with a high piezoelectric coefficient and a high dielectric constant which can be applied in energy-harvesting and energy conversion. Recently, Parangusan et al. [21] studied the P(VDF-HFP)/ZnO nanofibers as piezoelectric nanogenerators. They reported that the crystalline β-phase of P(VDF-HFP) increases with the proportional increase in ZnO filled in the P(VDF-HFP) matrix. The dielectric permittivity of pure P(VDF-HFP) nanofibers is 8 and that of another P(VDF-HFP)/ZnO nanofiber is 38. It exhibits an output voltage of 2.8 V.

This research aimed to study the phase and structure of electrostrictive P(VDF-HFP)/ZnO nanofibers enhance their electromechanical performance, which was prepared by the electrospinning method. Their surface topography, elemental analysis, phase structure, and dynamic mechanical analysis can be performed by using SEM, XRD, FTIR, and DMA techniques, respectively. LCR meter and tensile testing were then used to evaluate the dielectric constant and Young’s modulus as a function of the filler concentration, respectively. Moreover, the electrostrictive behavior, the figure of merit, and the energy-harvesting capability of P(VDF-HFP)/ZnO nanofibers are innovations and are worth focusing on. Finally, the potential for energy-harvesting applications of those electrostrictive nanofibers was assessed as an aspect of nanogenerators and nano-electronic devices in the future.

## 2. Experiment

### 2.1. Materials and Preparation

The pellets P(VDF-HFP) were purchased from Solef 11010/1001, Solvay Solexis. P(VDF-HFP) was the matrix and ZnO was the filler. The ZnO nanoparticles in the size range of 22–25 nm were purchased from the College of Nanotechnology; KMITL, Thailand. The solvent was the *N*,*N*-dimethylformamide (DMF) which was purchased from Sigma Aldrich (Fluka^®^ 40255).

**Figure 1:**
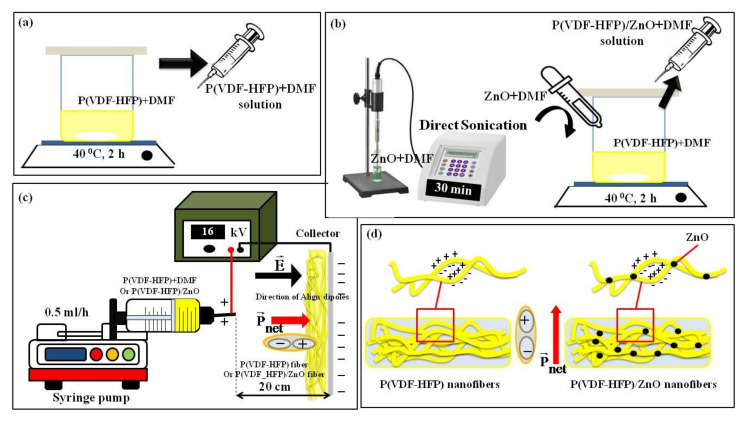
Schematic drawing of the samples’ preparation: (**a**) homogeneous P(VDF-HFP) solution; (**b**) homogeneous P(VDF-HFP)/ZnO solution; (**c**) the electrospinning setup; and (**d**) P(VDF-HFP) nanofibers and with ZnO nanoparticles.

The pure P(VDF-HFP) solution was fabricated by the addition of 5 g P(VDF-HFP) pellets to 20 mL of DMF and stirred for 2 h at 40 °C. The solution was heated and continuously stirred until homogeneous, as shown in Figure 1a. The undoped ZnO (0 wt%) and Co-doped ZnO (5, 10, 15, and 20 wt%) nanopowders were dispersed in the same solvent mixture (5 mL) and then sonicated for 30 min to disperse the filler. The dispersed filler solution was mixed with each of the P(VDF-HFP) solutions, and the whole mixture was magnetically stirred for 2 h to obtain a homogeneous mixture. The P(VDF-HFP)/ZnO solution was kept in a glass bottle for 24 h to remove some bubbles in the solution. Finally, those solutions were used for the electrospinning process, as shown in Figure 1b.

Figure 1c shows the electrospinning setup to obtain the pure P(VDF-HFP) nanofiber and composite nanofibers. The viscous solution was loaded in a 20 mL plastic syringe connected to a stainless-steel needle. The pure P(VDF-HFP) and its nanocomposite solution were electrospun at a flow rate of 0.5 mL/h with a syringe pump (Nz1000 NEWERA Pump Systems Inc., New York, NY, USA). The dimension of the tip (positive pole) to a collector (negative pole) was fixed as 20 cm and the applied voltage was 16 kV using a high voltage supply (PHYWE Item no. 13671-93, Germany). Ultimately, the pure and P(VDF-HFP)/ZnO composite nanofibers were obtained on an aluminum foil substrate pasted on the collector. A thickness gauge was used to measure the thickness of all samples.

### 2.2. Characterization

#### 2.2.1. Surface Topography

The morphology, dimension, and adhesion of the fillers on composite nanofibers were investigated by scanning electron microscope (SEM, FEI Quanta 400, The Netherlands). The average diameter of fibers was diagnosed from the SEM image using image processing software (ImageJ; National Institutes of Health, 1.46, MD, USA). High voltage and magnification of 20 kV and 10 k, respectively, were used to process the specimens.

#### 2.2.2. Elemental Analysis

The energy dispersive X-ray (EDX) microanalysis is a technique of elemental analysis associated with electron microscopy based on the generation of characteristic X-rays that reveals the presence of elements present in the specimens. The spectrum of EDX microanalysis contains both semi-qualitative and semi-quantitative information. The EDX technique can be considered a useful tool to detect nanoparticles in composite nanofibers that require the elemental determination of samples. The EDX (X-stream-2, Oxford, UK) was used analysis an elemental of the samples. The sample was observed by the signals of element mapping. The mapping images showed the chemical components in the surface, such as fluorine (F), carbon (C), and Zinc (Zn).

#### 2.2.3. Crystalline Structure and Phase Investigation

The crystalline electrospun co-doped ZnO and undoped ZnO nanoparticles were investigated by an X-ray diffractometer (XRD; X’Pert MPD, Philips, The Netherlands) with Cu-K_α_ radiation (= 0.154 nm) conducted at 30 mA and 40 kV in the 2θ range of 5–90° at a scanning rate 0.05°/1 s. The percentage crystalline degree $\left(X_{c}\right)$ of the sample can be calculated according to Equation (1) [26]:

(1) $$X_{c}=\frac{\Sigma A_{Cr}}{\Sigma A_{Cr}+\Sigma A_{amr}}\times 100\%$$

where $\Sigma A_{Cr}$ and $\Sigma A_{amr}$ are the integral area of crystalline and amorphous diffraction peaks, respectively. Furthermore, as regards phase structure, the α-phase and β-phase contents in the samples were interpreted by Fourier transform infrared spectrometer (FTIR; Vertex70, Bruker, Germany). The FTIR spectra of samples in the range of 400–4000 cm^−1^ were reported. The fraction of the β-phase $F\left(\beta \right)$ was given by using the Lambert–Beer law which is given as [27]:

(2) $$F\left(\beta \right)=\frac{A_{\beta }}{\left(\frac{k_{\beta }}{k_{\alpha }}\right)A_{\alpha }+A_{\beta }}=\frac{A_{\beta }}{1.26A_{\alpha }+A_{\beta }}$$

where $A_{\beta }\;$and $A_{\alpha }$ are the absorption peaks of the crystalline phase at 840 and 763 cm^−1^, respectively. $k_{\beta }\left(7.1\times 10^{4}\;cm^{2}mol^{-1}\right)$ and $k_{\alpha }\left(6.1\times 10^{4}\;cm^{2}mol^{-1}\right)$ are the absorption coefficients at the respective wavenumber. The number 1.26 is the ratio of absorption coefficients at 763 and 840 cm^−1^, respectively. The overall β contents in the different shapes were compared by calculating the absolute β fraction (%β), defined as the sample of $F\left(\beta \right)$ and$\;X_{c}$, as in the equation [28]:

(3) $$\%\beta =F\left(\beta \right)\times X_{c}$$

#### 2.2.4. Dynamic Mechanical Analysis

The mechanical properties of the specimens have three items, namely the storage modulus ($E^{\prime }$); the loss modulus (E); and the tan delta (tanδ). The sample’s elastic behavior can be detected with the storage modulus. The tan delta is a ratio of the loss modulus to storage modulus. It is often called damping and it is a gauge of the energy dissipation of material. The β-relaxation, which was assigned to the segmental motion within the amorphous phase, is detected at −50 °C. The α-relaxation is checked above room temperature. This relaxation is the factor that is mainly responsible for the elastic properties of the material and for the short-term creep behavior [29]. All of this can be identified by a dynamic mechanical analysis (DMA, Perkin Elmer) apparatus at a heating rate of 5 °C/min. The DMA testing was operated under isochronal conditions (frequency 1 Hz), from −100 to 140 °C. The amplitude of the dynamic stress which was small enough to ensure a linear viscoelastic response from the samples was 0.1 MPa.

#### 2.2.5. Mechanical Properties

The strain gauge setup was used to determine the elastic modulus of the sample. The nanofiber sheet samples (80 mm thickness) were cut to 5 mm wide and 30 mm long shape. The fixed end of the sample was clamped to a force sensor, while the other end was clamped to a linear motor moving stage (RC ROBOCylinder Model RCP 2CR-SA6, 220 mm-long). It was stretched in the length direction to a 5% strain maximum at an elongation rate of 5 mm/min. The elongation vs. force curves were resolved for all samples, and converted to strain and stress, whilst the Young’s modulus was calculated from the slope of strain and stress curves.

#### 2.2.6. Electrical Properties

The dielectric constant ($\varepsilon_{r}$), loss tangent (tan δ), and electrical conductivity (σ) of nanofibers were evaluated as a function of filler concentration and frequency in a range of 1–10^5^ Hz by the LCR meter (IM 3533 HIOKI, Japan) under room conditions. A voltage of 1 V was applied to the specimens in sweep testing mode. The area of the electrodes of the sample is $1.96\times 10^{-5}{\;m}^{2}$ and the same thickness of 200 ± 100 μm. Dielectric permittivity, loss tangent and electrical conductivity were determined by the value of parallel capacitance and conductivity readings, respectively.

#### 2.2.7. Electrostriction

**Figure 2:**
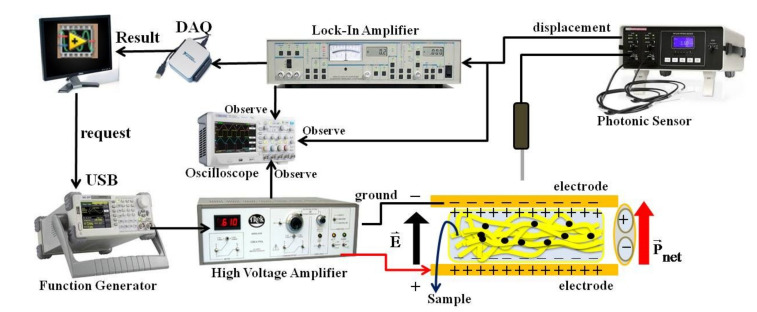
Setup used for measuring the deformation of the sample at low frequency.

The effect of ZnO nanoparticles on the electrostrictive behavior of P(VDF-HFP) composites nanofibers was examined by measuring the deformation at a low frequency and an electric field (E) strength-induced strain (S) with a photonic displacement apparatus (MTI-2100 Fotonic sensor, New York, NY, USA, sensitivity range of 5.8 µm/V), as demonstrated in Figure 2. All fibers were accessed as approximately 200 ± 50 μm-thick strands; they were sandwiched between two brass electrodes and the weight of the top brass disc was 5 g, which was advisable for small stress to avoid the clamping of the sample. The electric field-induced strain was applied along the thickness direction. The thickness deformation of all samples was calculated by the photonic sensor and a lock-in amplifier (Trek model 610E, New York, NY, USA).

#### 2.2.8. Energy Conversion Ability

**Figure 3:**
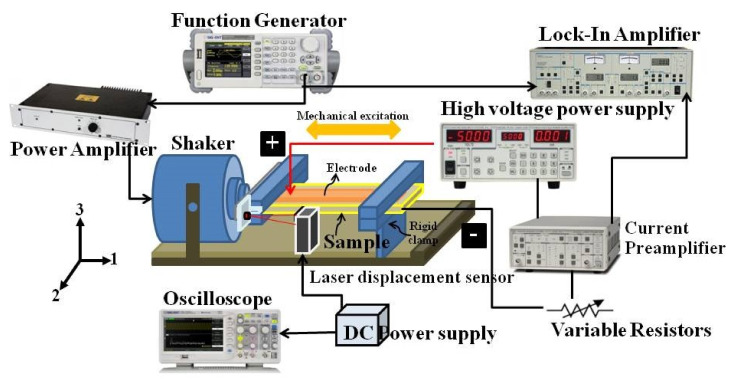
The setup for measuring the current induced in the electrostrictive nanofibers.

Figure 3 demonstrated the energy-harvesting setup for measuring the current induced in the samples. The rectangular-shaped sample was sandwiched between two copper tape electrodes. The right and left ends of the sample were fixed to a rigid camp and the shaker, respectively. The mechanical shaker, which originated from a power amplifier and a function generator, vibrated at 20 Hz excitation in the horizontal sample direction. The displacement of the sample was observed by a laser displacement sensor (KEYENCE IA030, Japan). The DC bias electric field was generated by a high-voltage power supply applied through the copper tape on the sample surface. The sample and the bias voltage source were connected through a variable resistor to determine an optimal load resistor ($R_{C}$). The induced current in the sample was measured by a current preamplifier, and a lock-in amplifier monitored it. The maximum power harvested ($P_{max}$) was calculated using $P2_{C}{}_{max}$ where *I* is the induced current from the sample.

## 3. Results and Discussion

### 3.1. Surface Morphology and Elemental Analysis

**Figure 4:**
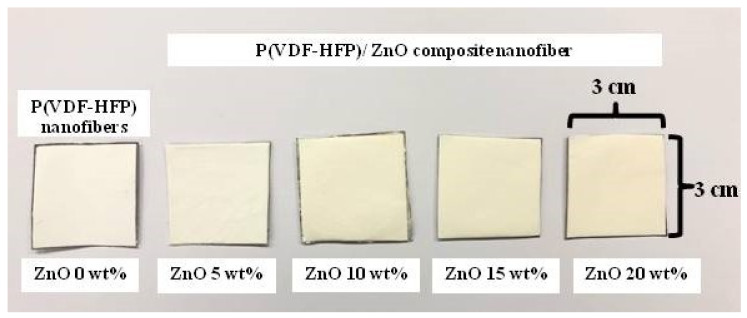
A photograph of specimens for P(VDF-HFP) nanofibers and P(VDF-HFP)/ZnO composite nanofibers (5, 10, 15, and 20 wt%).

A photograph of the specimens for the pure P(VDF-HFP) nanofiber, P(VDF-HFP)/ZnO composites nanofiber at 5, 10, 15, and 20 wt%, respectively, is presented in Figure 4. It shows that as the concentration of ZnO increases, the color of the composite fibers changes from a white to yellowish color. The formation of the yellow color indicates the presence of additives. 

**Figure 5:**
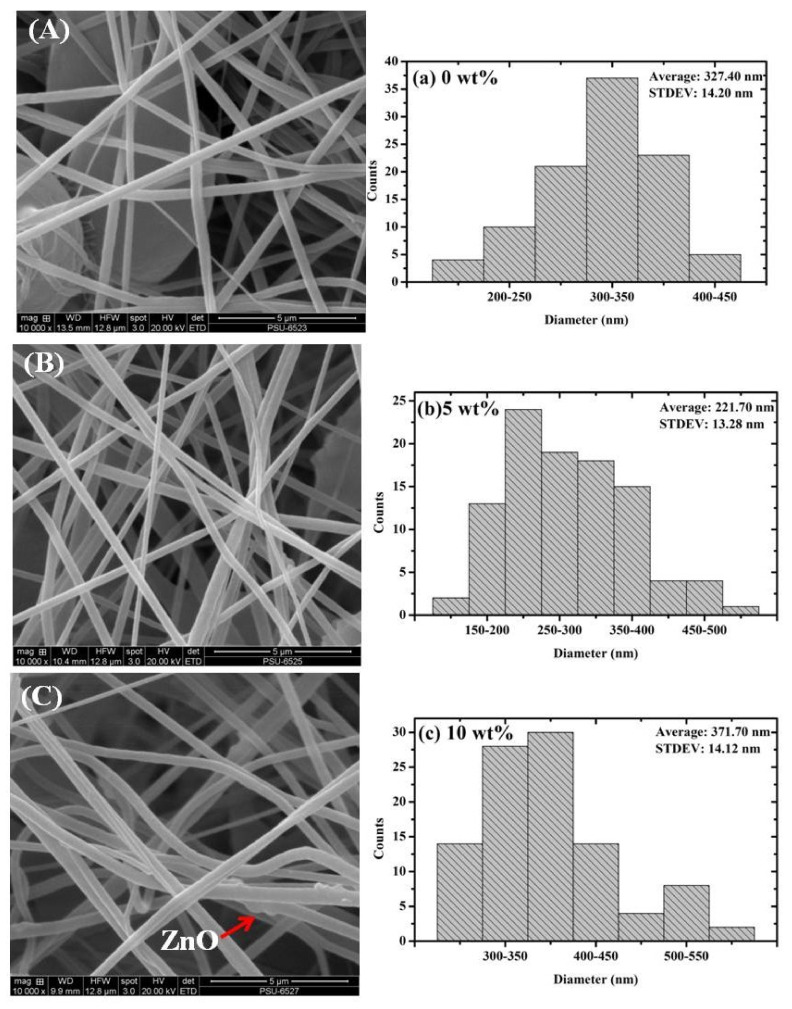

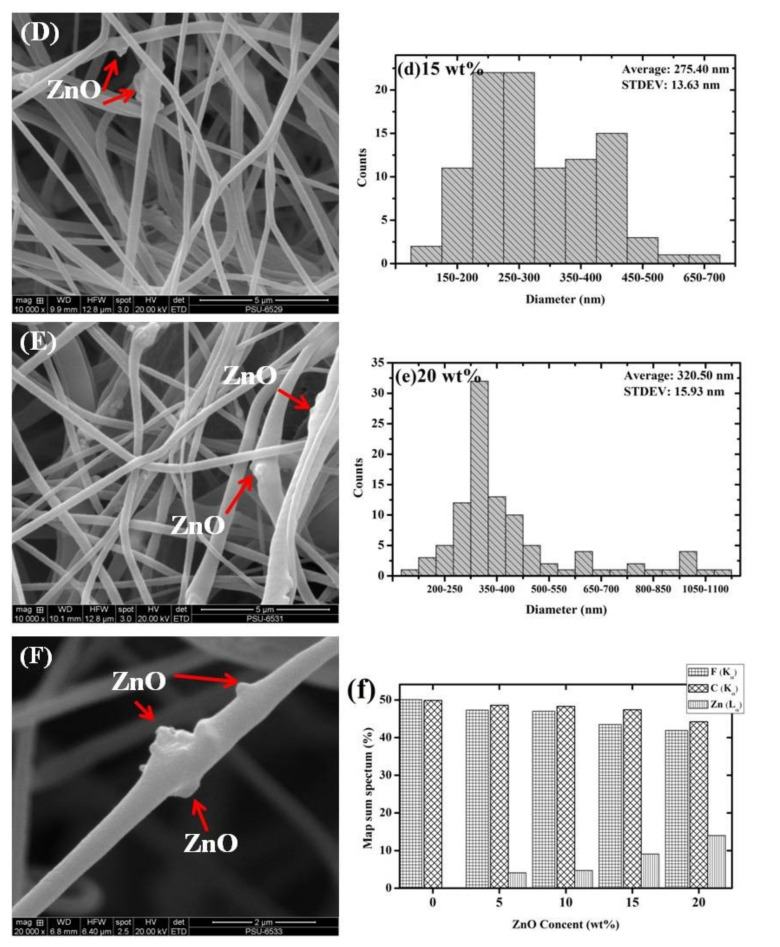
The morphology and diameter distribution statistics of nanofiber: (**A** and **a**) P(VDF-HFP) nanofiber; (**B** and **b**) P(VDF-HFP)/ZnO 5 wt%; (**C** and **c**) P(VDF-HFP)/ZnO 10 wt%; (**D** and **d**) P(VDF-HFP)/ZnO 15 wt%; (**E** and **e**) P(VDF-HFP)/ZnO 20 wt%; (**F**) zoom in ZnO on the surface of a P(VDF-HFP)/ZnO 20 wt% nanofiber; and (**f**) elemental mass composition of ZnO in the composite P(VDF-HFP)/ZnO nanofibers.

The SEM image for pure P(VDF-HFP) nanofibers is shown in Figure 5A. We investigated whether nanofibers had a smooth, uniform in diameter, and beat-free nanofibers, indicating that the condition applied in the electrospinning process was suitable. The average width was 327.40 ± 14.20 nm (Figure 5a). As shown in Figure 5B–F, the exterior morphological of P(VDF-HFP)/ZnO composites nanofibers exhibited a rough surface, non-uniformity in terms of width, and increased the fiber diameter distribution. The average fiber diameter was unstable in the range of 210–380 nm (Figure 5b–e). The results also indicated that an increase in ZnO heterojunction causes the width of the nanofibers to increase. The dimension of the nanofiber’s increased is due to the effect of the viscosity of the solution. The dope viscosity may be changed by raising the concentration of the polymer or by the inclusion of the insoluble filler. This could be because the tension on nanofibers during fabrication increases by increasing the density of ZnO. Moreover, the elemental mass composition of the P(VDF-HFP) nanofibers pure, and ZnO-loaded nanofibers were observed by EDX and the repercussion is demonstrated in Figure 5f. The electrospun nanofibers produced the creation of an open three-dimensional network, which presents a large surface area-to-value ratio and flexible surface functionalities that can promote intense contact with the charge distribution for the actuators’ application.

### 3.2. Crystalline Structure and Phase Investigation

**Figure 6:**
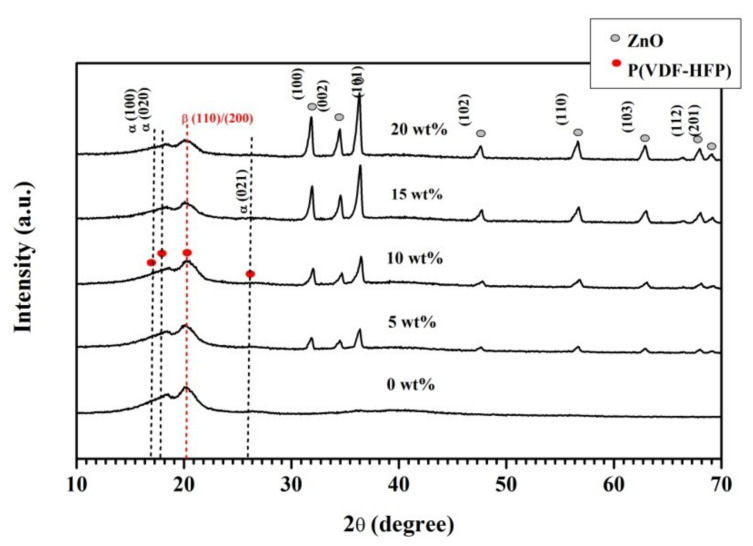
XRD analysis of P(VDF-HFP) nanofibers pure and P(VDF-HFP)/ZnO composite nanofibers (5, 10, 15, and 20 wt%).

Figure 6 showed the crystalline phases of the pure and P(VDF-HFP)/ZnO nanofibers with various weight percentages of ZnO (5, 10, 15, and 20 wt%). The crystallinity of fragments was considered by analyzing the X-ray diffraction pattern. The semi-crystalline P(VDF-HFP) polymer showed characteristic reflections of the crystalline phases at 2θ = 17.9° (020) and 26.8° (021), which relates to the non-polar α-phase crystals while 2θ = 18.5° (100) and 20.1° (110) correspond to the γ-phase crystals which co-exist with the α-phase. The characteristic peaks at 2θ = 20.3° (110) and (200) and 36.7° (020) and (100) correspond to β-phase diffraction [30]. The characteristic peaks with a hexagonal structure of ZnO were assigned at 2θ = (31.75°, 34.44°, 36.25°, 47.54°, 56.56°, 62.87°, 67.92°, and 69.06°), which was determined for the diffractions of the (100), (002), (101), (102), (110), (103), (112), and (201) [31]. The diffraction peak of ZnO incorporated in the P(VDF-HFP) nanofibers indicated the presence of ZnO. The pure P(VDF-HFP) and composites fibers showed a strong β peak (110) at 2θ = 20.3° for the β-phase having an all-trans (TTTT) conformation. The pure P(VDF-HFP) exhibits the peak at 20.3° but there were more peaks in P(VDF-HFP)/ZnO nanofibers which were strong peaks at 31.75°, 34.44°, and 36.25° as shown by the hexagonal wurtzite structure of ZnO. This means that the the crystallinity was increased by the addition of ZnO nanoparticles and 20 wt% ZnO in P(VDF-HFP)/ZnO nanofibers exhibited the same peaks at 31.75°, 34.44°, and 36.25°, but with high intensity. It was observed that the addition of ZnO enhanced the β-phase for P(VDF-HFP)/ZnO nanofibers. This issue was similarly reported by Hemalatha Paranhusan et al. [21], who fabricated the P(VDF-HFP)/ZnO composite film which was enhanced by the β-phase crystallinity. Moreover, the diffraction peaks correspond to Co-doped ZnO nanoparticles were also observed in the range of 30–60° for the P(VDF-HFP)/Co-ZnO nanocomposites. It is consummated from the XRD pattern that the addition of the Co-doped ZnO nanofiller enhanced the β-phase formation in the P(VDF-HFP) nanofibers.

**Figure 7:**
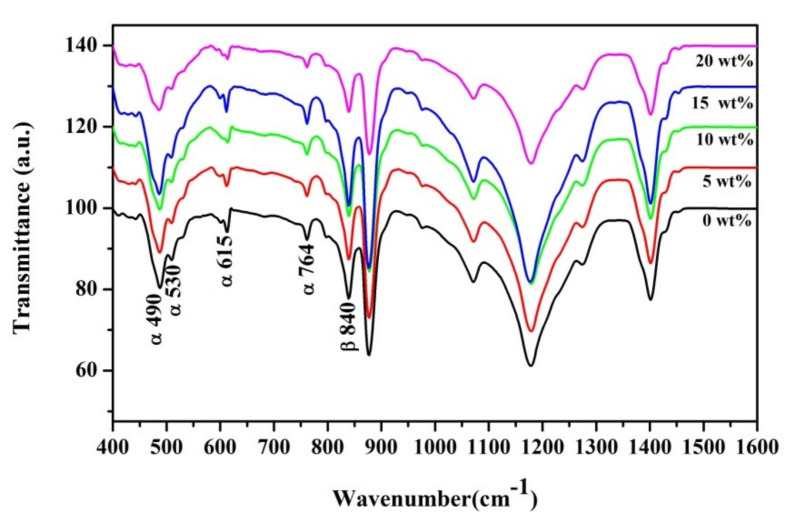
Infrared measurements for the P(VDF-HFP) and P(VDF-HFP)/ZnO electrospun membranes with different concentrations of ZnO (0, 5, 10,15, and 20 wt%), respectively.

Figure 7 illustrates the FTIR spectra of the pure P(VDF-HFP) and P(VDF-HFP)/ZnO nanofibers. In the literature [32], the transmittance bands in the IR spectra around 490 cm^−1^ (−CF_2_ wagging), 530 cm^−1^ (−CF_2_ bending), 615 cm^−1^ (skeletal bending), 764 cm^−1^ (−CF_2_ bending), 795 cm^−1^ (−CH_2_ rocking), and 975 cm^−1^ (twisting) referred to the non-polar α-phase of P(VDF-HFP). The great transmittance peaks of the β-phase, which was the electroactive polar β-polymorph with a parallel dipole moment, were found at 840 cm^−1^ (−CH_2_ rocking, −CF_2_ stretching, and skeletal C−C stretching). In all samples, the transmittance peaks for the α-phase were met while the absorbance peaks at 490 cm^−1^, 530 cm^−1^, and 840 cm^−1^ were important, implying a strong emergence of the electroactive β-phase. Various analyzers have reported that the surface charges on the Co-doped ZnO nanofillers collaborate with the molecular dipoles (CH_2_ or CF_2_) of P(VDF-HFP) and enhance the β-phase capacity of the composites. This can also be described based on positive charges that are present in the Co–ZnO nanofillers that interact with –CH_2_– dipoles of the P(VDF-HFP) segments in the nanocomposites.

Table 1 presented the crystallinity and the β-phase fraction in the crystalline of the samples. The crystallinity $X_{c}$ within the pure P(VDF-HFP) and composites is calculated by Equation (1) following the XRD results. The $X_{c}$ increased from 47.35 to 62.35% with the increasing concentration of ZnO. Moreover, the increase in crystallinity may be due to the interactions of the surfaced with the composites nanofibers chains, inducing the formation of the polar β-phase from non-polar α-phase. The results of the fraction of the β-phase, $F\left(\beta \right)$, was calculated by equation (2) using the Lambert–Beer law. The pure P(VDF-HFP) nanofibers had $F\left(\beta \right)$ ~ 72.72%. Furthermore, the $F\left(\beta \right)$ of P(VDF-HFP)/ZnO composite nanofibers increased from 72.72 to 76.03% when the concentration of ZnO increased. This result confirms the positive influence of semiconductor nanofillers on the β-phase formation, as previously reported. Parangusan et al. [21] reported that the increasing concentration of ZnO nanofillers from 0 to 2 wt% increased the β-phase content in P(VDF-HFP)/ZnO nanofibers from 37.5 to 54.6%. The absolute β fraction (%β) was calculated by Equation (3). The results indicate that the (%β) of the incorporation of P(VDF-HFP) with 5, 10, 15, and 20 wt% ZnO was larger than the pure P(VDF-HFP) of 40.04, 42.57, 45.17, and 47.40%, respectively. The issue of the electroactive β-phase was clearly enhanced by adding with the ZnO nanofiller and the electrospinning process which was confirmed by XRD patterns and FTIR spectra.

### 3.3. Mechanical Properties

**Figure 8:**
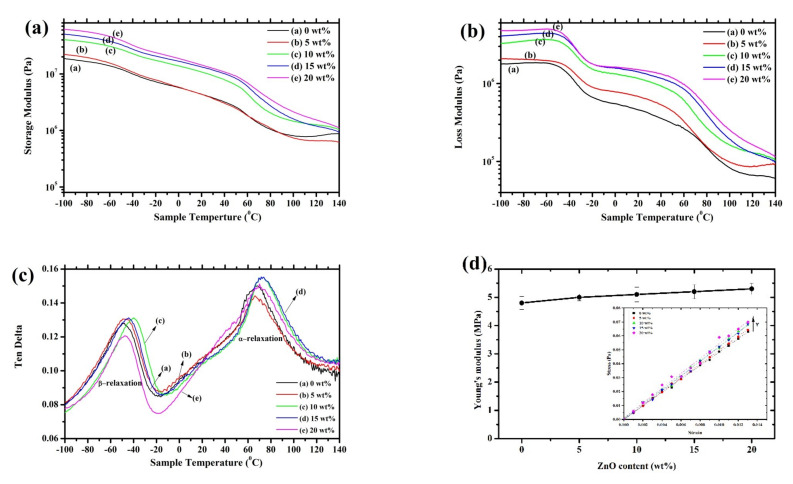
The dynamic mechanical analysis curves of pure P(VDF-HFP) fiber and composites fiber as a function of temperature for the (**a**) storage modulus; (**b**) loss modulus; (**c**) tan delta; and (**d**) Young’s modulus of the samples versus the weight fraction of ZnO; the slope of stress–strain curves of all samples is shown as an inset.

The thermomechanical properties and the glass transition temperatures can be analyzed by using dynamic mechanical analysis (DMA). Figure 8a–c display the storage modulus (E’), the loss modulus, and the tan delta of fabricated composite nanofibers. The storage modulus decreases with increasing temperature. The storage modulus in both the rubbery and glassy regions increased due to the concentration of ZnO increasing from 0 to 20 wt%. The high storage modulus of the composites P(VDF-HFP)/ZnO 20 wt% at low temperature ~−100 to −50 °C confirms the reinforcement effect at the molecule interfaces. This can be attributed to the restricted molecular mobility in the P(VDF-HFP)/ZnO nanofibers by the strengthened interaction with the matrix of the polymer [33]. A slow decrease in E’ is observed from −40 to 0 °C, which was ascribed to the glass transition of P(VDF-HFP) [34]. This result showed that semiconductor nanofillers influenced the glass transition temperature of the P(VDF-HFP) nanofibers.

Tan delta, as a function of temperature for the pure P(VDF-HFP) and P(VDF-HFP)/ZnO composites nanofibers, is presented in Figure 8c. A β-relaxation evaluate in tanδ was observed at T ≈ −45 °C for the P(VDF-HFP)/ZnO fiber 15 wt%. It was then assigned to cooperative segmental motions of the leading chains in amorphous regions. The β-relaxation was attached to dynamic glass transition in amorphous and other semi-crystalline polymers. The damping combined with the β process implies that it is comparatively high for the fiber with a broad β-transition from −80 to −20 °C and a maximum at −45 °C. Generally, the glass transition temperature (Tg) of a polymeric material was determined from the peak of the Ten delta curve [31]. The Ten delta has a peak at approximately −40 °C assigned to the glass transition of pure PVDF. The glass transition temperatures are approximately −47, −48, −40, −45, and −46 °C for a pure P(VDF-HFP) fiber, and 5, 10, 15, and 20 wt% of ZnO, respectively. Above 25 °C, a new relaxation process emerges, being more pronounced in terms of Tan delta for samples. This action, labeled α, is combined with disturbances in the crystalline fraction [27]. This higher-T_g_ relaxation is also found in a variety of flexible semi-crystalline polymers, and its mechanism could be similar to the one in P(VDF-HFP). The α-relaxation is influenced by the solid-state rheological features of P(VDF-HFP) above room temperature.

Figure 8d shown the mechanical moduli of undoped and P(VDF-HFP)/ZnO composites nanofibers with various concentrations of ZnO which were calculated from the slope of the stress–strain curves (as an inset). It was found that the tensile modulus slightly increases with increase in the concentration of ZnO nanoparticles, and in accordance with the results performed by DMA analysis, which was previously mentioned. In general, all composites’ systems presented an increase in Young’s modulus for the polymer matrix filled with the filler according to mixing law, Young’s modulus increases with the increasing amount of filler loading. Young’s modulus is an important parameter influencing the M_33_ coefficient and was therefore determined in tensile mode for all samples. Moreover, Young’s modulus can explain the linear elastic behavior of the material. The elastic modulus often increased after ZnO loading due to the rule of mixing properties. Obviously, the modulus slightly increases in a very low filler concentration. Several articles explain that the conductive filler concentration is mainly due to an increase in the interfacial surface area between the filler particles and the host matrix [35,36]. After that, Young’s moduli tend to be constant for a higher aspect ratio of ZnO (upon the low concentration condition to keep the low Young’s modulus) in the P(VDF-HFP) matrix. This result could be attributed to the poor level distribution of filler as revealed by SEM topographic images. The Young’s modulus of the composites is quite constant for a higher aspect ratio of ZnO under the low concentration conditions.

### 3.4. Electrical Properties

**Figure 9:**
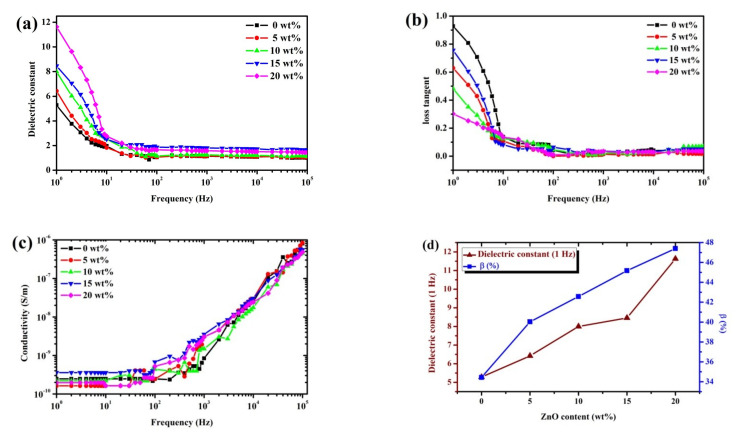
Room temperature frequency-dependent (**a**) dielectric constant; (**b**) dielectric loss; (**c**) AC conductivity of P(VDF-HFP)/ZnO nanocomposites with 0, 5, 10, 15, and 20 wt% ZnO content, respectively; and (**d**) the effect of the ZnO content on the dielectric constant and absolute β fraction.

The dielectric constant (*ε_r_*), loss tangent (tan δ), and conductivity (σ) as functions of the frequency with various content of P(VDF-HFP) filled with 0–20 wt% ZnO were shown in Figure 9a–c. In Figure 9a, the dielectric constant has its maximum at a low frequency and it decreased with increasing frequency. At a low frequency, the dielectric constant is associated with free dipoles that vibrate in an alternating field. There is an orientational polarization of polar groups as well as space charge polarization. Therefore, the dielectric constant is normally heightened with the space charge distribution in the P(VDF-HFP)/ZnO composites nanofibers. It is achievable that the ZnO nanoparticles embedded in the polymer matrix acted as sources of space charge in the P(VDF-HFP) matrix. The dielectric constant increase with ZnO content at an operating frequency of 1 Hz. The maximum dielectric constant is 11.63 at 1 Hz for electrospun P(VDF-HFP)/ZnO 20 wt%. Clearly, the ZnO reduced air gaps and added surface charges causing strong Maxwell–Wagner interfacial polarization. At a high frequency, the dipoles can no longer follow the rapid changes in the field direction. The dielectric constant decreased rapidly to 100 Hz and then reduced slowly after that due to a lack of adequate time to polarize the dipoles. Figure 9b displayed the dielectric loss (tan δ) which decreased with the applied frequency. Significant dielectric losses are caused by the charges at low frequencies (1 Hz), which induced relaxations due to the polarization effects caused by interfacial polarization and dipole orientation. Furthermore, it was presented that the loss tangent decreased with the addition of ZnO in the P(VDF-HFP) matrix due to the increased dielectric constant and also relates to decreased dielectric loss. The smallest dielectric loss is 0.3 at 1 Hz for P(VDF-HFP)/ZnO 20 wt% nanofibers. Figure 9c shows the alternating current (AC) conductivity, which linearly increased with frequency, indicating that the number of charge carriers also increased. The observed development in electrical conductivity may be attributed to the polarization of bound charges [9,37]. The inclusion of semiconductor filler probably increased the charge density and this might be attributed to the conductive networks formed by the surface contacts in the fibrous matrix, as well as the free electrons in it [24,38]. The dielectric constant heightened with the fraction of ZnO at an operating frequency of 1 Hz and the increased number of absolute β fractions (%β), affecting both the electrical and mechanical properties shown in Figure 9d. The dielectric constant depending on the crystalline fraction in the polymer [39], the improved crystallinity was leading to sizeable interfacial polarization. The dielectric constant was enhanced in the composites compared to pure P(VDF-HFP). The dielectric constant of the composites increased up to a critical loading of ZnO, similar to the reported dielectric behavior of PVDF composites containing multiwalled carbon nanotubes [16]. This study indicates that the P(VDF-HFP)/ZnO nanofibers are promising electrostrictive materials for actuation and can be fabricated with an easy preparation.

### 3.5. Electrostriction Behavior

**Figure 10:**
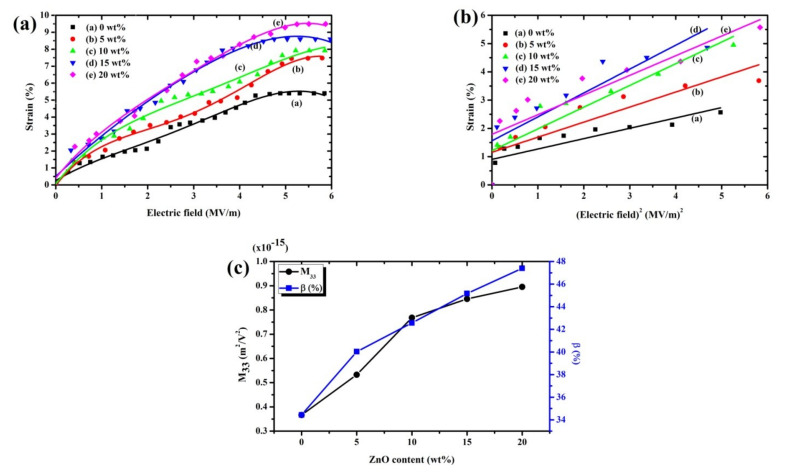
The longitudinal strain in the P(VDF-HFP)/ZnO composites nanofibers at 1 Hz: (**a**) strain as a function of the electric field; (**b**) strain as a function of E_3_^2^; and (**c**) the effect of the ZnO content on the electrostrictive coefficient and the absolute β fraction.

Figure 10a depicts the longitudinal strain (S_33_) behavior for all samples which was induced by an external electric field (E_3_) at a frequency of 1 Hz. The total thickness strain has an almost quadratic relation to the applied electric field. It can be demonstrated by electrostriction which was shown on $S_{33}=M_{33}E_{3}^{2}$ (M_33_ is the electrostrictive coefficient) and Maxwell stress [40]. The Maxwell stress effect involved electrostatic attractions and interactions with the charges on the electrodes shown on $S_{M}=\varepsilon_{0}\varepsilon_{r}E_{3}^{2}/Y$. Here, ε_0_ is the permittivity of free space, *ε_r_* is the permittivity, and Y is Young’s modulus. According to a prior publication, the strain from the Maxwell stress is minimal compared to the measured strain, and therefore the Maxwell stress effect can be neglected [41]. Hence, it can be assumed that the measured strain was only due to the electrostrictive effect. The electric field-induced strain increases when increasing the content of ZnO. Though, when the external electrical field expands above approximately 4.5 MV/m, the electrostrictive strain becomes saturated due to the saturation of the electric field-induced polarization [41]. Figure 10b shows that the relation of the strain (S_33_) versus the square of the applied electric field (E^2^_3_) due to the slope equals the electrostrictive coefficient (M_33_). It is wholly presented that the electrostriction of composites nanofibers is significantly heightened when filling ZnO. The M_33_ increase with the fraction of ZnO wherewith increased of the dielectric constant and absolute β fraction can play a crucial role, affecting both the mechanical and electrical properties presented in Figure 10c. This is reflected by the abruptly increased space charge distribution, and the increasing dielectric constant was increased with the ZnO content. Table 2 displayed the dielectric constant at 1 Hz, Young’s modulus and electrostrictive coefficients for all cases that remarkably increased with ZnO loading. This result indicates that the ZnO component effectively enhanced the electrostriction behavior of P(VDF-HFP)/ZnO composites nanofibers. Moreover, it has been reported that the improved electrostriction relates to the dielectric constant and Young’s modulus of the composites [37,40]. They proposed that the electrostrictive coefficient (M_33_) is almost proportional to $\varepsilon_{0}\varepsilon_{r}/Y$. The M_33_ coefficient was increased with the dielectric constant of composite nanofibers when increasing ZnO filler, while Young’s modulus slightly increases with loading. Comparison of the M_33_ coefficient and the dielectric constant of all samples was presented in Table 2. By assuming that the modulus is kept constant, the M_33_ coefficient of nanofibers is directly proportional to $\varepsilon_{0}\varepsilon_{r}$. Several previous studies [33,42] have acknowledged that the electromechanical properties of a nanocomposite strongly depend on the nanofiller content, nanoparticle size, and relative permittivity. Thus, the dispersion of the filler in a fiber matrix is very important, not only depending on the loading, particle size, and the interfacial surface area [43]. Therefore, an increase in the specific interfacial area is essential to try and increase the dielectric constant and a fiber form product will have a larger surface area to volume ratio as well as flexibility. In our case, the permittivity increase with ZnO content suggests an increase in the interfacial charges and electrostrictive coefficient. Moreover, this study indicates that the P(VDF-HFP)/ZnO composites nanofibers may represent the promising route for obtaining the electrostrictive composites nanofibers for actuation, employing ZnO nanoparticles.

### 3.6. Energy-Harvesting Performance

**Figure 11:**
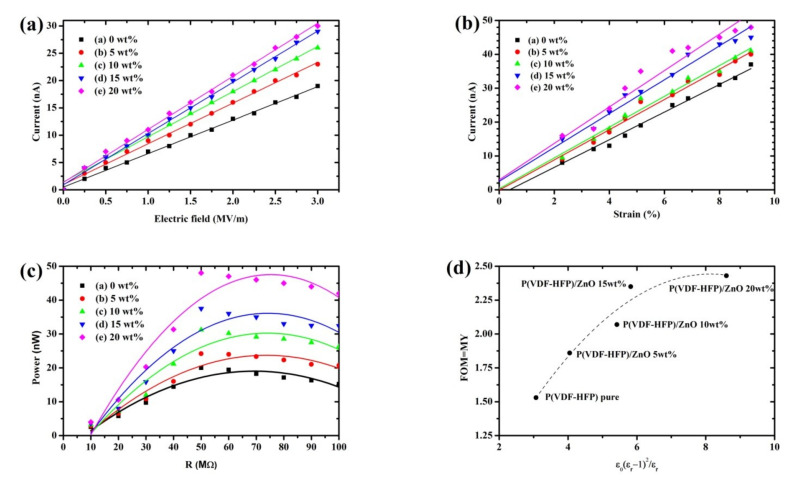
(**a**) The output current as a function of the electric field at 5% strain; (**b**) the output current as a function of %strain at E = 3 MV/m; (**c**) the variation of the electric power output as a function of load resistance at E = 3 MV/m; and (**d**) the figure of merit (FOM) as a function of $\varepsilon_{0}{\left(\varepsilon_{r}-1\right)}^{2}/\varepsilon_{r}$ for all samples at 20 Hz.

Figure 11a shows the output current in all samples under the electric field strength and strain rate (E = 0 to 3 MV/m, S = 5%) at an operating frequency of 20 Hz by the clamp–clamp setup which is shown in Figure 3. In all cases, the output current increases with the increasing electric field. At an electric field of 3 MV/m, the output current increases with the increasing ZnO content. The output current was increased when the dielectric constant was increasing, which was caused by the interface charge density when the ZnO nanoparticles were scattered in the P(VDF-HFP) matrix. The highest harvested current was accessed with the P(VDF-HFP)/ZnO 20 wt%, whereas the lowest one was collected with the pure P(VDF-HFP) nanofibers—the difference between these being two-fold. Figure 11b shows the effect of %strain at an electric field of 3 MV/m with an output current of pure and composites samples. This result shows that output current increases with increasing %strain and output current also increases with an increasing ZnO content. The harvested electric power P = I^2^R is shown in Figure 11c as a function of load resistance (R), measured at 20 Hz frequency for the pure P(VDF-HFP) and the P(VDF-HFP)/ZnO composites nanofibers. In all these samples, the power at the beginning increased with the load resistance and then quickly decreased, so that the optimal resistance (R_c_) giving the maximal power was well defined. The optimal loads shifted towards lower values with ZnO content. This matches the reported relationship between the optimal load resistance and dielectric permittivity [42]. The output current and maximum power harvested at the optimal resistance increased with the ZnO concentration. Figure 11d demonstrates the figure of merit (FoM) = MY as a function of $\varepsilon_{0}{\left(\varepsilon_{r}-1\right)}^{2}/\varepsilon_{r}$ at 20 Hz. Its trend towards increasing with the ZnO loading is due to the changes in the dielectric constant, which increased with the filling. An increase in the electrostrictive coefficient leads to a higher FoM for our composites.

Table 3 shows the dielectric constant, the Young’s modulus, the electrostrictive coefficient (M_31_), and the power density of pure P(VDF-HFP) compared to other composites. The dielectric constant increase with ZnO content offers an increase in the interfacial charges. The Young’s modulus also increased with the ZnO content, did not have a significantly increased modulus. Several previous studies explained that the electrostrictive coefficient (M_31_) was calculated by [44,45]; $I=2\mathrm{MYE}\int \dot{S}\mathrm{dA}$. Here, it is the harvested current; Y is the Young’s modulus of the samples; E is the electric field; $\dot{S}$ is the strain rate; and A is an area of the electrode. The electrostrictive coefficient increases with the increasing concentration of ZnO. The ZnO effect in the dielectric constant, due to the interfacial charges in the composite, strongly affected the electrostrictive coefficient while effects on the modulus were weak in comparison. The P(VDF-HFP)/ZnO composites nanofibers show a high-power density compared to the pure P(VDF-HFP) nanofibers due to their high dielectric constant. This may be due to the interfacial interactions of ZnO fillers with the P(VDF-HFP) nanofiber matrix, as the filler nanoparticles may increase the interface charge.

## 4. Conclusions

In this research, we fabricated electroactive composite nanofibers with P(VDF-HFP)/ZnO. The ZnO content had a significant effect on the mechanical properties, electrical properties, electrostriction behavior, and energy-harvesting performance of P(VDF-HFP) nanofibers. The volume of ZnO can be enhanced by the β-phase fraction, interfacial charges, dielectric constant, and electrostrictive coefficient content in the P(VDF-HFP) nanofibers. In this work, the P(VDF-HFP)/ZnO 20 wt% composite nanofibers exhibited the better 47.40% absolute β fraction among the cases tested. Moreover, this case gave the highest 11.63 dielectric constants at 1 Hz and also had the largest electrostrictive coefficient M_33_ of approximately $0.89\times 10^{-15}$ m^2^/V^2^. The energy-harvesting performance of P(VDF-HFP)/ZnO 20 wt% was assessed through a figure of merit (FoM), of approximately 2.4, which was also consistent with the electrostrictive and dielectric properties. The composites nanofibers display the higher dielectric permittivity since a more significant β-phase fraction and the increase in a space charge distribution when increasing the ZnO fillers. These two contributions are related to the rise of the electrostriction coefficient, which can enhance the longitudinal strain versus the electric field of composites nanofibers. The maximum power density that 20 wt% ZnO in P(VDF-HFP) nanofibers gave 1201.25 nW/cm^3^ at 20 Hz. Therefore, ZnO nanoparticles distributed into electrostrictive P(VDF-HFP) nanofibers can provide large power density and an excellent efficiency of electromechanical conversion.

## Figures and Tables

**Table 1:** Analysis of the crystallinity and β-phase fraction in the crystalline of the samples.

Sample (wt%)	*X_c_* (*%*)	*F(β)* (*%*)	%*β*
P(VDF-HDP) Pure	47.35	72.72	34.43
P(VDF-HFP)/ZnO 5 wt%	53.42	74.94	40.03
P(VDF-HFP)/ZnO 10 wt%	56.81	75.73	42.57
P(VDF-HFP)/ZnO 15 wt%	59.52	75.89	45.17
P(VDF-HFP)/ZnO 20 wt%	62.35	76.03	47.40

**Table 2:** Dielectric constant (*ε_r_*), Young’s modulus, and M_33_ coefficient for the fabricated P(VDF-HFP)/ZnO composited nanofibers.

Sample (Nanofibers)	$\varepsilon_{r}$ 1 Hz	Y (MPa)	M_33_ (×10^−15^) (m^2^/V^2^) 2 V/μm, 1 Hz	$\frac{\varepsilon_{0}\varepsilon_{r}{}_{}}{Y}$ (×10^−11^)
P(VDF-HDP) Pure	5.28	4.8	0.37	0.97
P(VDF-HFP)/ZnO 5 wt%	6.42	5.0	0.53	1.14
P(VDF-HFP)/ZnO 10 wt%	8.00	5.1	0.77	1.39
P(VDF-HFP)/ZnO 15 wt%	8.46	5.2	0.84	1.44
P(VDF-HFP)/ZnO 20 wt%	11.63	5.3	0.89	1.94

**Table 3:** The dielectric constant ($\varepsilon_{r}$), the Young’s modulus (Y), the M_31_ electrostrictive coefficient, and the power density for the synthesized P(VDF-HFP)/ZnO composites nanofibers, shown with P(VDF-TrFE-CFE) composites films included for comparison.

Sample (Nanofibers)	$\varepsilon_{r}$ 20 Hz	Y (MPa)	M_31_ (20 Hz) (×10^−18^) (m^2^/V^2^)	Power Density (nW/cm^3^)
P(VDF-HDP) Pure	1.31	4.8	3.19	500.00
P(VDF-HFP)/ZnO 5 wt%	1.37	5.0	3.73	605.00
P(VDF-HFP)/ZnO 10 wt%	1.87	5.1	4.05	781.25
P(VDF-HFP)/ZnO 15 wt%	2.08	5.2	4.51	937.50
P(VDF-HFP)/ZnO 20 wt%	2.20	5.3	4.58	1201.25

At 20 Hz, E = 3 V/μm, strain rate 1 S^−1^ in clamp–clamp setup.

## Data Availability

The data presented in this study are available on request from the corresponding author.
